# Bleeding Outcomes of Direct Oral Anticoagulants and Vitamin K Antagonists for Acute Venous Thromboembolism: A Cross-Sectional Study

**DOI:** 10.7759/cureus.60616

**Published:** 2024-05-19

**Authors:** Zeeshan Javaid, Shakeel Ahmad Awan, Muhammad Babar, Imran Khawaja, Zia Qamar

**Affiliations:** 1 Hematology, South Tees Hospitals NHS Foundation Trust, Middlesbrough, GBR; 2 Acute Medicine, University Hospitals of Derby and Burton NHS Foundation Trust, Derby, GBR; 3 Internal Medicine, University Hospitals Birmingham NHS Foundation Trust, Boston, GBR; 4 Internal Medicine, Ayub Teaching Hospital, Abbottabad, PAK; 5 Pulmonology, Ayub Teaching Hospital, Abbottabad, PAK

**Keywords:** anticoagulants, major organ bleeding, vka, doac, vte

## Abstract

Background: Venous thromboembolism (VTE) is a widespread and significant cause of morbidity and mortality on a global scale. The primary objective of this cross-sectional study is to examine the impact of anticoagulant therapy on major organ hemorrhage events in patients diagnosed with acute venous thromboembolism (VTE). Specifically, this research compares the effects of vitamin K antagonists (VKAs) and direct oral anticoagulants (DOACs).

Materials and methods: This retrospective observational study examined the medical records of 46 patients who had been diagnosed with VTE and were receiving treatment with DOACs or VKAs. The documentation of patient characteristics encompassed demographic information, comorbidities, and treatment particulars. Within 30 days of hospital admission, the incidence of significant organ bleeding events, with an emphasis on gastrointestinal and intracranial hemorrhage, was the primary outcome evaluated.

Results: Overall, 46 patients with VTE who were treated with oral anticoagulation therapy participated in the study. Twenty-four and 22 patients were administered VKAs and DOACs, respectively. The similarity in baseline characteristics between the DOAC and VKA groups ensured that the analyses were well-matched. The examination of bleeding sites unveiled subtle variations, as the DOAC group exhibited a progressive increase in the incidence of intracranial bleeding (12, 55.5%), while the VKA group demonstrated a surge in upper gastrointestinal bleeding (12, 50%) as well. While lacking statistical significance, these observed patterns are consistent with prior research that indicates that DOACs may have a lower risk of catastrophic hemorrhage in comparison to VKAs. The overall in-hospital mortality rate for patients treated with VKA was 33.3% (n=8), while that treated with DOAC was 18.2% (n=4). These differences did not reach statistical significance (P>0.05). In a similar vein, the evaluation of mortality associated with hemorrhage revealed six (25%) in the group receiving VKA and three (13.6%) in the group receiving DOAC; the P value was not statistically significant (P>0.05).

Conclusions: This study contributes valuable insights into bleeding outcomes associated with anticoagulant therapy for acute VTE. The nuanced differences in bleeding patterns highlight the complexity of anticoagulant selection, emphasizing the importance of considering bleeding site considerations. The comparable mortality rates support existing evidence regarding the favorable safety profile of DOACs.

## Introduction

Globally, venous thromboembolism (VTE) is a prevalent condition, affecting an estimated one in every 12 individuals aged 45 and older [1]. High-risk patients may experience a mortality rate of 10%-30% within one month due to VTE [2]. Patients frequently experience prolonged hospital stays due to the condition's associated mortality risk, which places an additional social and economic strain on the healthcare system [3]. The annual economic impact of venous thromboembolism (VTE) can differ significantly across countries, potentially reaching tens of billions of euros [4]. Asians have a significantly lower incidence of venous thromboembolism (VTE) than their Western counterparts, with rates estimated to be around 15%-20% of those observed in the West. However, recent years have seen a substantial increase in the prevalence of venous thromboembolism (VTE) among the Asian population, primarily attributed to developments in diagnostic methods and heightened awareness of the condition [5,6].

The conventional approach has been to administer low-molecular-weight heparin (LMWH) followed by vitamin K antagonists (VKAs) for an extended period of time. In recent years, direct oral anticoagulants (DOACs) have witnessed a significant increase in both popularity and accessibility. In lieu of vitamin K antagonists (VKAs), the current recommendations for the management of venous thromboembolism (VTE) in 2021 suggest that direct oral anticoagulants (DOACs) be used for both the initial and secondary treatment of VTE [7]. In the context of acute venous thromboembolism (VTE) management, direct oral anticoagulants (DOACs) and vitamin K antagonists (VKAs) have been compared. Two meta-analyses of randomized controlled trials (RCTs) [8,9] found that DOACs were associated with a decreased risk of fatal bleeding, severe bleeding, and any hemorrhage in patients with VTE when compared to VKAs. On the contrary, an alternative study [10] found that DOACs were correlated with an increased risk of stroke among patients with antiphospholipid syndrome (APS) when compared to VKAs. Concerning patients with cerebral venous thrombosis, DOACs and VKAs exhibited comparable safety and efficacy [11]. A retrospective observational study [12] found that DOACs were associated with earlier and higher rates of thrombus resolution and fewer adverse events in patients with left ventricular thrombus (LVT) compared to VKAs. A critical decision, the selection of an anticoagulant regimen must strike a balance between safety and efficacy. This study examines the incidence of major organ hemorrhage in patients receiving DOACs and VKAs for the treatment of venous thromboembolism (VTE), with a particular focus on intracranial and gastrointestinal bleeding. Comparing the incidence of major organ hemorrhage (intracranial and gastrointestinal) in patients receiving DOACs and VKAs for the treatment of VTEs is the purpose of this study.

## Materials and methods

Study design

This retrospective observational study examines the medical records of patients who were prescribed DOACs and VKAs for the treatment of venous thromboembolism (VTE) from 20-01-2019 to 20-04-2019 (IBR approval number: ATH00756793). The clinical progression and results were recorded until the 30th day following the patient's admission to the hospital.

The institutions that took part in the study were those that maintained interdisciplinary teams available around the clock to manage hemorrhage associated with anticoagulation in their intensive care units and emergency departments. Approval of the study protocol was obtained from every pertinent institutional ethics committee.

Patients

Patients who experienced significant hemorrhage during treatment with VKAs or DOACs were enrolled in the research. For hemorrhage classification, the modified definition of the International Society on Thrombosis and Haemostasis (ISTH) for non-surgical patients was utilized. Major bleeding was defined as the occurrence of fatal bleeding, bleeding in a critical location or organ, or hemoglobin loss of at least 2 mg/dL (1.24 mmol/L) [13].

Inclusion criteria

The sample includes patients with a confirmed diagnosis of VTE irrespective of their age. All the patients included are with complete medical records, including treatment details and bleeding events.

Exclusion criteria

We excluded patients with contraindications to anticoagulant therapy and incomplete or missing medical records or patients on multiple anticoagulant regimens simultaneously.

Data collection

Patient demographics, comorbidities, medication adherence, and follow-up duration were recorded from the treatment files of the patients. The primary outcome is the incidence of major organ bleeding events and in-hospital mortality within 30 days of admission.

Statistical analysis

The Statistical Package for the Social Sciences (SPSS) version 26 (IBM SPSS Statistics, Armonk, NY) was used for data analysis. Baseline characteristics of VKA and DOAC groups were compared using independent t-tests and chi-square/Fisher's exact tests for continuous and categorical variables, respectively. Major organ bleeding events were assessed with chi-square/Fisher's exact tests, including subgroup analyses for specific bleeding sites. In-hospital mortality rates and bleeding-associated mortality were calculated and compared using chi-square/Fisher's exact tests, with statistical significance set at P<0.05.

## Results

The study included 46 patients with severe hemorrhage during oral anticoagulation therapy. The comparison of patient characteristics between the vitamin K antagonist (VKA) and direct oral anticoagulant (DOAC) groups revealed no significant differences. Both groups exhibited similar mean ages (VKA: 64.04±5.51; DOAC: 64.64±6.47), weights (VKA: 70.62±12.74; DOAC: 69.36±11.59), HAS-BLED scores (score to assess the risk of bleeding) (VKA: 2.75±0.44; DOAC: 2.81±0.39), and CHA2DS2-VASc scores (score to determine the risk of stroke) (VKA: 4.70±0.46; DOAC: 4.68±0.48). Additionally, there were no significant differences in gender distribution, type of index event, level of consciousness, need for mechanical ventilation, occurrence of hemorrhagic shock, and other baseline characteristics (Table 1). These baseline similarities underscore the comparability of the VKA and DOAC groups, setting the foundation for evaluating the primary outcomes related to major organ bleeding incidence.

**Table 1:** Baseline sample characteristics (N=46) VKA, vitamin K antagonist; DOAC, direct oral anticoagulant; CHA2DS2-VASc score, score to determine the risk of stroke; HAS-BLED score, score to assess the risk of bleeding; HTN, hypertensive intracranial hemorrhage; CKD, chronic kidney disease; DM, diabetes mellitus; PE: pulmonary embolism

Patient characteristics	VKA group (n=24)	DOAC group (n=22)	P value
Age	64.04 (±5.51)	64.64 (±6.47)	0.66
Weight	70.62 (±12.74)	69.36 (±11.59)	0.375
HAS-BLED score	2.75 (±0.44)	2.81 (±0.39)	0.845
CHA2DS2-VASc score	4.70 (±0.46)	4.68 (±0.48)	0.575
Gender			
Male	13 (54.2%)	12 (54.5%)	0.555
Female	11 (45.8%)	10 (45.5%)	
Type of index event			
Venous thromboembolism	13 (54.2%)	8 (36.4%)	0.204
Pulmonary embolism	5 (20.8%)	10 (45.5%)	
Both VTE and PE	6 (25.0%)	4 (18.2%)	
Level of consciousness			
Unconscious	12 (50.0%)	6 (27.3%)	0.281
Arousable	8 (33.3%)	10 (45.5%)	
Awake	4 (16.7%)	6 (27.3%)	
Mechanical ventilation	13 (54.2%)	15 (68.2%)	0.331
Hemorrhagic shock	12 (50.0%)	15 (68.2%)	0.211
Trauma	3 (12.5%)	4 (18.2%)	0.592
Antiplatelet agents	4 (16.7%)	5 (22.7%)	0.605
Nonsteroidal anti-inflammatory drugs	21 (87.5%)	16 (72.7%)	0.207
DM	17 (70.8%)	16 (72.7%)	0.887
HTN	14 (58.3%)	14 (63.6%)	0.713
CKD	5 (20.8%)	4 (18.2%)	0.821

**Figure 1:**
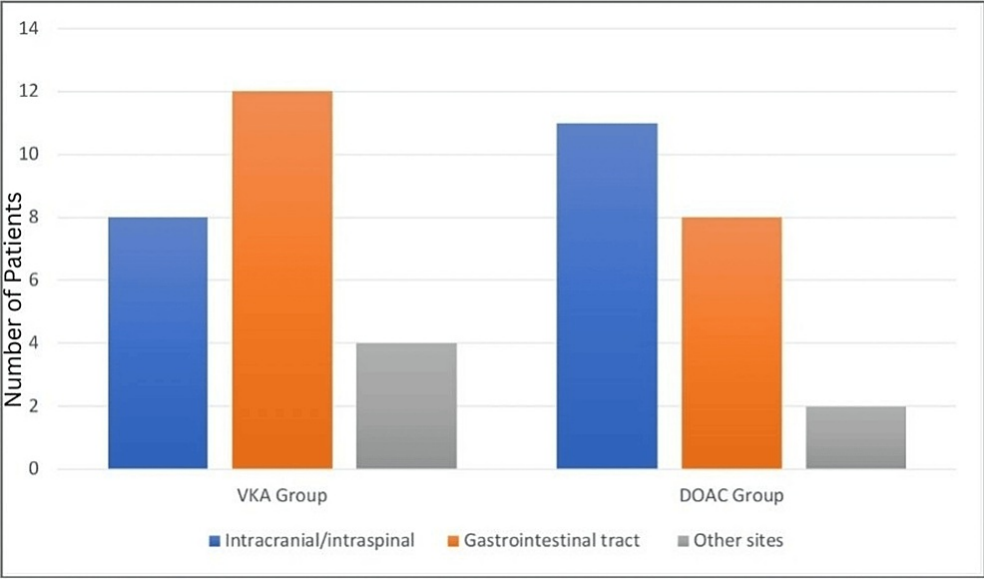
Major organ bleeding events among groups The graph shows the number of patients on the vertical axis and two groups VKA and DOAC on the horizontal axis. VKA group: The graph indicates that eight patients among 24 patients of the VKA group experienced intracranial/ intraspinal bleeding, which is 33.3% of the total patients. Similarly, 12 out of 24 patients had gastrointestinal tract bleeding, which makes up 50% of the total patients, and four patients out of 24 had other types of bleeding, which makes up 13.6%. DOAC group: According to the graph, 11 patients among 22 patients had intracranial/ intraspinal bleeding, which shows 50% of the total patients in the group. Similarly, eight patients out of 22 experienced gastrointestinal tract bleeding, which reflects 36.4% of the group, and three out of 22 patients had other bleeding events, which is 13.6% of the total patients in the group. VKA, vitamin K antagonist; DOAC, direct oral anticoagulant

The results for major organ bleeding events in patients receiving different treatments (VKA and DOAC) indicate varying patterns in the distribution of bleeding locations. In the VKA group, eight (33.3%) patients experienced intracranial/intraspinal bleeding, 12 (50%) had gastrointestinal tract bleeding, and four (16.7%) had other types of bleeding. Comparatively, in the DOAC group, 11 (50%) patients had intracranial/intraspinal bleeding, eight (36.4%) experienced gastrointestinal tract bleeding, and three (13.6%) had other bleeding events (Figure 1).

Comparison of bleeding sites in VKA (n=24) and DOAC (n=22) groups revealed that six (25%) and eight (36.4%), respectively, had intracranial/intraspinal bleeding, with specific breakdowns of two (8.3%) and four (18.2%) for intracerebral bleeding. Upper gastrointestinal bleeding occurred in eight (33.3%) VKA patients and four (18.2%) DOAC patients. Despite variations, no statistically significant differences in bleeding site distribution were observed between the two anticoagulant groups (P>0.05) (Table 2).

**Table 2:** Comparison of bleeding sites VKA, vitamin K antagonist; DOAC, direct oral anticoagulant

Bleeding site	VKA group (n=24)	DOAC group (n=22)	P value
Intracranial/intraspinal			
Intracerebral	6 (25.0%)	8 (36.4%)	0.550
Subdural	2 (8.3%)	4 (18.2%)	
Subarachnoid	2 (8.3%)	-	
Epidural	-	1 (4.5%)	
Gastrointestinal tract			
Upper	8 (33.3%)	4 (18.2%)	0.68
Lower	2 (8.3%)	2 (9.1%)	
Intramuscular	1 (4.2%)	2 (9.1%)	0.73
Retroperitoneal	1 (4.2%)	-	0.61
Intraocular	2 (8.3%)	1 (4.5%)	0.54

The investigation into 30-day overall in-hospital mortality among patients yielded rates of 33.3% (n=8) in the VKA group and 18.2% (n=4) in the DOAC group; despite variations, no statistically significant differences were observed (P>0.05). Similarly, the assessment of bleeding-associated mortality demonstrated rates of 25% (n=6) in the VKA group and 13.6% (n=3) in the DOAC group, with a non-significant P value of 0.46 (Table 3).

**Table 3:** In-hospital mortality within 30 days of admission VKA, vitamin K antagonist; DOAC, direct oral anticoagulant

Variables	VKA group (n=24)	DOAC group (n=22)	P value
30-day overall in-hospital mortality	8 (33.3%)	4 (18.2%)	0.32
Bleeding-associated mortality	6 (25%)	3 (13.6%)	0.46
Other causes of death	2 (8.3%)	1 (4.5%)	0.10
Respiratory failure	1 (4.1%)	-	0.62
Acute heart failure	1 (4.1%)	1 (4.5%)	0.45

## Discussion

In the current study, comparable baseline characteristics of patients receiving VKAs and DOACs highlight the successful establishment of well-matched groups. This ensured that subsequent analyses on bleeding outcomes were not confounded by significant demographic or clinical differences.

An in-depth exploration of bleeding sites further illuminated the nuanced differences between VKAs and DOACs. While there were no statistically significant variations, the trends suggest that DOACs may be associated with a higher incidence of intracranial bleeding, particularly intracerebral and subdural, compared to VKAs. Upper gastrointestinal bleeding was more prevalent in the VKA group. The absence of statistical significance might be attributed to the study's sample size, warranting consideration in future research endeavors. The observed differences in bleeding patterns align with meta-analyses indicating that DOACs are associated with a reduced risk of fatal bleeding compared to VKAs [14]. Notably, the higher incidence of intracranial bleeding in the DOAC group is consistent with findings in patients with antiphospholipid syndrome [15]. These nuanced variations underscore the complexity of anticoagulant selection in diverse clinical scenarios. Our findings resonate with the existing literature, emphasizing the importance of bleeding site considerations. The prevalence of upper gastrointestinal bleeding in the VKA group aligns with known safety profiles [16]. However, the impact of comorbidities, such as cirrhosis, cannot be disregarded. The absence of statistical significance may be attributed to sample size constraints.

The assessment of 30-day overall in-hospital mortality demonstrated comparable rates between VKAs and DOACs, with mortality risk nominally higher in VKA-treated patients with major bleeding. However, statistical analysis showed no significant difference. The comparable mortality rates between VKAs and DOACs within 30 days of admission corroborate with existing evidence [17,18]. These results are congruent with the meta-analyses that highlighted DOACs' favorable safety profile in terms of bleeding outcomes [9].

The observed inconsistencies in the in-hospital mortality rates in our current study, notably higher at 33.5% (n=8) in the VKA group and 18.2% (n=4) in the DOAC group, compared to recent cross-sectional studies, warrant careful consideration. In contrast to our findings, comparable mortality rates were reported in two notable studies: a prospective observational study (17.5% for VKA-treated patients) and the "Canadian study" (15.2% for VKA-treated patients) [19,20]. Similarly, the mortality rates for DOAC-treated patients demonstrated remarkable consistency across studies, with rates of 8.3% and 9.8% in the respective studies. Moreover, a recently conducted multicenter study addressing the same research theme revealed lower (18% versus 33.5% for VKA and 9% versus 18.2% for DOAC) mortality rates as compared to the current study findings [21].

Several factors may contribute to the observed disparities. First and foremost, differences in patient populations, including variations in demographics, comorbidities, and baseline health status, could influence mortality outcomes. Our study might encompass a distinct patient cohort with unique risk factors or disease characteristics, contributing to the observed variations in mortality rates. Second, variations in healthcare practices, treatment protocols, and regional disparities could impact patient outcomes. Differences in healthcare infrastructure, access to medical care, and treatment adherence may all play roles in influencing mortality rates. It is imperative to acknowledge the limitations inherent in cross-sectional studies, including the potential for selection bias and the inability to establish causal relationships. The nature of our study design, while providing valuable insights, may introduce certain limitations that impact the generalizability of our findings.

## Conclusions

The bleeding sites suggested potential differences, with DOACs showing a higher incidence of intracranial, intracerebral, and subdural bleeding compared to VKAs. Upper gastrointestinal bleeding was more prevalent in the VKA group. Despite variations in bleeding patterns, the 30-day overall in-hospital mortality rates and mortality risk in VKA-treated patients with major bleeding did not reach statistical significance, which can be due to the small sample size.
